# Magnetic Oculomotor Prosthetics for Acquired Nystagmus

**DOI:** 10.1016/j.ophtha.2017.05.028

**Published:** 2017-10

**Authors:** Parashkev Nachev, Geoff E. Rose, David H. Verity, Sanjay G. Manohar, Kelly MacKenzie, Gill Adams, Maria Theodorou, Quentin A. Pankhurst, Christopher Kennard

**Affiliations:** 1 Institute of Neurology, University College London, London, United Kingdom; 2 Orbital Clinic, Moorfields Eye Hospital NHS Foundation Trust, London, United Kingdom; 3 National Institute for Health Research Biomedical Research Centre, Institute of Ophthalmology, Bath Street, London, United Kingdom; 4 Department of Clinical Neurology, University of Oxford, Oxford, United Kingdom; 5 Strabismus Clinic, Moorfields Eye Hospital NHS Foundation Trust, London, United Kingdom; 6 Healthcare Biomagnetics Laboratory, University College London, London, United Kingdom

**Keywords:** D, diopter, PD, prism diopter

## Abstract

**Purpose:**

Acquired nystagmus, a highly symptomatic consequence of damage to the substrates of oculomotor control, often is resistant to pharmacotherapy. Although heterogeneous in its neural cause, its expression is unified at the effector—the eye muscles themselves—where physical damping of the oscillation offers an alternative approach. Because direct surgical fixation would immobilize the globe, action at a distance is required to damp the oscillation at the point of fixation, allowing unhindered gaze shifts at other times. Implementing this idea magnetically, herein we describe the successful implantation of a novel magnetic oculomotor prosthesis in a patient.

**Design:**

Case report of a pilot, experimental intervention.

**Participant:**

A 49-year-old man with longstanding, medication-resistant, upbeat nystagmus resulting from a paraneoplastic syndrome caused by stage 2A, grade I, nodular sclerosing Hodgkin's lymphoma.

**Methods:**

We designed a 2-part, titanium-encased, rare-earth magnet oculomotor prosthesis, powered to damp nystagmus without interfering with the larger forces involved in saccades. Its damping effects were confirmed when applied externally. We proceeded to implant the device in the patient, comparing visual functions and high-resolution oculography before and after implantation and monitoring the patient for more than 4 years after surgery.

**Main Outcome Measures:**

We recorded Snellen visual acuity before and after intervention, as well as the amplitude, drift velocity, frequency, and intensity of the nystagmus in each eye.

**Results:**

The patient reported a clinically significant improvement of 1 line of Snellen acuity (from 6/9 bilaterally to 6/6 on the left and 6/5–2 on the right), reflecting an objectively measured reduction in the amplitude, drift velocity, frequency, and intensity of the nystagmus. These improvements were maintained throughout a follow-up of 4 years and enabled him to return to paid employment.

**Conclusions:**

This work opens a new field of implantable therapeutic devices—oculomotor prosthetics—designed to modify eye movements dynamically by physical means in cases where a purely neural approach is ineffective. Applied to acquired nystagmus refractory to all other interventions, it is shown successfully to damp pathologic eye oscillations while allowing normal saccadic shifts of gaze.

An estimated 0.24% of the population has persistent nystagmus, an abnormal rhythmic oscillation of the eyes typically of neural origin.^1^ When acquired later in life, nystagmus often is associated with oscillopsia, a distressing perception of constant movement of the visual scene that is both intrinsically disabling and a cause of reduced visual acuity.^2^ The impact of nystagmus on life is substantial, and visual functioning scores typically are lower than those for better-known visual impairments such as age-related macular degeneration.^3^ Although many drugs have been evaluated, no generally effective pharmacologic treatment exists.^4^ Given the heterogeneity of the underlying physiologic dysfunction, no single agent conceivably could target what is likely to be a complex multiplicity of potential neural loci, and thus the historical wide individual variability in the response to pharmacotherapy is likely to continue.

Although nystagmus has many different origins within the central nervous system, all converge on a single target: the extraocular muscles that move the globe itself. Therefore, an intervention that attenuates the action of oculomotor muscles could offer an effective treatment for nystagmus, regardless of the cause. Unfortunately, although strabismus surgery may sometimes improve the location of the point of gaze where the nystagmus is less pronounced—known as a *null point*—complete immobilization of the eyes generally is unhelpful because it blocks any ability to shift gaze; this type of intervention generally is restricted only to cases of third-nerve palsy with very large angle deviations refractory to other surgical approaches.^5^

The ideal solution is to attenuate the oscillation of the eyes when the patient is fixating a target, while still permitting saccadic movements necessary to refixate. Because saccadic forces are substantially greater than the forces generating the pathologic drift that triggers each nystagmic jerk,^6^ it follows that an intermediate force could damp nystagmus without preventing saccades. Crucially, such force needs to be delivered remotely, without direct physical contact that otherwise would lead to postoperative fibrosis and loss of ocular motility. Currently, the only practical means of achieving action at a distance is magnetic, permanent, rare earth magnets having sufficient magnetic flux density while remaining of implantable size.

We therefore conceived a permanent magnetic implant with a pair of interacting elements: an orbital part fixed to the orbital wall and an ocular part tethered to an extraocular muscle. The implant location is determined by the plane of the dominant drift component of the nystagmus—the orbital floor for vertical nystagmus and the lateral wall for horizontal—with the ocular part sited within the Tenon's sheath of the relevant muscle, thereby maintaining a mobile tissue boundary between the 2 elements. The magnetic force thus damps ocular oscillation, while leaving a full range of movement and essentially normal saccadic gaze shifts. Herein we describe the design, implantation, and evaluation of a prototype device in 1 patient with highly symptomatic, treatment-resistant upbeat nystagmus and report the clinical observations, including those from a more than 4-year follow-up period after surgery.

## Methods

### Patient

The patient, a right-handed man in full-time employment as a driver of commercial heavy goods vehicles, was referred to the neuro-ophthalmology clinic at 49 years of age for progressive unsteadiness, slurred speech, vertigo, and variable diplopia evolving over 2 years. At presentation, there was upbeat nystagmus on lateral gaze, but not in the primary position, and broken pursuit. Horizontal diplopia was present just for distance causing a concomitant distance right esotropia of 8 prism diopters (PD) base out. His Snellen visual acuity was 6/5+3 in the right eye and 6/5–3 in the left. Other than mild dysarthria, the rest of the neurologic examination was unremarkable.

Routine investigations revealed a mediastinal mass subsequently identified as stage 2A, grade I, nodular sclerosing Hodgkin's lymphoma, for which he received 3 courses of standard doxorubicin, bleomycin, vinblastine, and dacarbazine chemotherapy and involved field radiotherapy of 35 Gy in 20 fractions, leaving him in sustained, complete remission. There were otherwise no biochemical, hematologic, serologic, or cerebrospinal fluid abnormalities. Magnetic resonance imaging of the brain showed only mild cerebellar atrophy.

Despite successful tumor treatment, his symptoms progressed to include distressing oscillopsia. Repeat examination now showed nystagmus in the primary gaze with no null point, and his visual acuity had dropped to 6/9 bilaterally. The angle of squint increased and stabilized at 30/35 PD base out, near and distance. Ocular motility revealed mild lateral gaze defects (−0.5) in both eyes, −0.5 right inferior rectus underaction, and an A pattern. This symptomatic progression contributed to his loss of employment.

A diagnosis of paraneoplastic cerebellar syndrome was made. There was no response to corticosteroid immunomodulation or to a course of intravenous immunoglobulins. The clinical picture remained static until the present intervention, 7 years after the original diagnosis.

In the intervening years, many pharmacologic agents for symptomatic treatment of the nystagmus were tried without success, including benzodiazepines, carbamazepine, valproate, levetiracetam, gabapentin, acetazolamide, phenytoin, memantine, baclofen, 3-4 diaminopyridine, and 4-aminopyridine.

Faced with symptoms he found profoundly debilitating, the patient asked us to consider novel experimental approaches. This led us to recollect an informal report from a Brazilian ophthalmologist, Harley Bicas, about the successful use of implantable magnets in nystagmus (theoretically elaborated by him after our intervention was concluded^7^). The patient's request prompted the research project described in this study. Informed consent was obtained following procedures approved by an ethics committee with specific expertise in handling device studies (London-Dulwich NRES Committee). All research adhered to the tenets of the Declaration of Helsinki.

### Implant Design

We designed a 2-part rare-earth magnetic implant consisting of a small cylindrical ocular part designed to be sutured to an extraocular muscle near its insertion and within the tendinous sheath and a larger cylindrical orbital part designed to be fixed to the orbital wall. The thickness of the orbital magnet was conceived to be variable, guided by the external testing of prototypes of the same diameter, but of different lengths, externally before implantation (see below). The ocular magnet was designed to be made of sintered samarium–cobalt material, and the orbital magnet was designed to be made of sintered neodymium–iron–boron, with the magnetic axis in both being aligned to the cylindrical axis. The ocular magnet was a cylinder 3 mm in diameter and 1 mm in length, and the orbital magnet was a cylinder 3.73 mm in diameter and 2 mm in length. Because permanent magnetic materials are biologically reactive, each part was encased in grade 2 titanium, with laser welded joints, and the titanium cases included small flanges to facilitate suturing (for the ocular part) or gluing (for the orbital part). The final design was custom manufactured by Magnet Sales & Service Ltd (Swindon, United Kingdom). Note that the magnetic materials exhibit sufficient temperature stability to allow standard sterilization techniques to be applied.

### Extraoperative Procedures

#### Oculometrics

The capacity to maintain fixation at cardinal positions of the gaze was assessed with the aid of high-resolution video-based eye tracking. A single, white, square-shaped stimulus subtending 0.5° of visual angle was displayed on a black background on a 21-inch cathode ray tube monitor (1024 × 768 pixels; refresh rate, 140 Hz) at a viewing distance of 60 cm. Eye position was monitored at 1000 Hz (EyeLink 1000; SR Research, Ottawa, Canada). Both pupil and corneal reflections were used (dual tracking mode), and eye position calibration was performed using EyeLink's standard 9-point calibration screen.

The patient followed the fixation stimulus sequentially to each of 9 positions on the screen in a randomized predetermined order while keeping his head fixed with the aid of a chinrest and headrest. Each fixation point was maintained for 30 seconds. The 9 positions fell on an invisible regular rectilinear grid subtending 30° of visual angle in each dimension. He was encouraged to maintain fixation as accurately as he could. No optical correction was used. Data for each eye were acquired during both binocular and monocular viewing; binocular data are reported as closest to real-life experience. Both the raw position data with respect to time and saccades detected by the EyeLink built-in algorithm were collected. The EyeLink criteria for a saccade were acceleration of 1000° per second squared, velocity of 30° per second, and amplitude of more than 0.1°. This experimental set up was replicated identically for both preoperative and postoperative testing.

Oculometric data were analyzed independently for each recording session, eye, and gaze location using custom Matlab scripts (Mathworks, Natick, MA). For each rapid eye movement detected by EyeLink as a saccade according to the above criteria, we calculated the displacement amplitude (in degrees) and the slow-phase drift velocity in the interval 100 to 20 ms preceding it (in degrees per second). The interval between each rapid movement gave us a measure of instantaneous frequency (in hertz), and the product of frequency and amplitude gave us the derived metric of intensity (in degrees hertz), which has been used as a real-life informative measure of the severity of nystagmus.^8^ Because each of these parameters can be derived for every nystagmic jerk, we were able to perform statistical comparisons, separately for each parameter, across each recording session, each eye, and each of the 9 gaze locations. We used a 2-sample Kolmogorov-Smirnov test for these comparisons, with asymptotic calculation of significance values. The medians of the distributions for each metric, session, eye, and gaze location were presented as spider plots, together with standard errors of the medians, with the asymptotic significance values of the relevant statistical comparison presented on each axis.

Although reasonable calibration was achieved, for robustness we performed an additional analysis of the amplitude and drift velocity parameters where the eye position data were subjected first to an affine transform, derived by minimizing the squared error over possible transformation matrices, that takes into account translational, rotational, skew, and scaling differences between calibrated and actual calibration positions. Because such off-line calibration correction inevitably assumes a linear interpolation of error across locations, unjustifiably propagating errors across the field of view, we present this analysis in addition to the conventionally calibrated data.

For illustrating the effect of the intervention on fixation stability, we estimated the spatial probability densities of the raw fixation position data (sampled at 1 ms) for the most stable location (lower middle) in each eye, both before and after surgery. The estimates were calculated using bivariate adaptive kernel density estimation.^9^ These plots index the degree of stability of the gaze by the width of the distribution: the narrower, the more stable the gaze. Note that no inferential comparison was performed here or was intended.

#### External Implant Testing

To confirm that the force exerted by the implant would be sufficient to attenuate the patient's nystagmus without impeding saccades, we created a mechanical platform for applying the same force externally. Drawing on an established approach for nontraumatic, noninvasive application of forces to the eye,^10^ we adapted a table-mounted conventional slit-lamp microscope (SM-2; Takagi Seiko Co, Ltd., Iwafune, Nakano-shi, Nagano-ken, Japan), attaching to the headrest a custom-made stereotactic frame (Integrated Technologies Ltd, Ashford, Kent, United Kingdom) that allowed us precisely to position different-sized prototypes of the orbital part of the implant in relation to the tested eye using an adjustable cylindrical sliding arm fixed to the frame. A set of custom-made, fenestrated, polymethyl methacrylate scleral contact lenses, molded to the patient's eyes (made by Ken Pullum; http://www.kenpullum.co.uk/) were adapted to allow cyanoacrylate adhesive fixation of a single prototype of the ocular part of the implant on the external apex. Adhesive also was applied to the magnet surface to simulate the spacing of intervening tissue between the 2 parts of the implant. Because there is less scope for variation in the size of the ocular magnet, this was kept as a single, fixed disc of 3 mm in diameter, 1 mm in thickness (Samarium–cobalt material). Tygon tubing (1.6 mm inner diameter, 3.2 mm outer diameter; Cole-Parmer Instrument Co Ltd), attached to the fenestration of each lens with cyanoacrylate adhesive, was linked to a manually operated 10-ml syringe through which a low-pressure suction adherence to the ocular surface could be maintained. Any force applied to the contact lens thus could be transmitted securely to the eye. The slit-lamp microscope allowed close ocular monitoring before, during, and after the procedure.

The patient was seated with his head on the slit-lamp headrest. Only the right eye was tested, because it was assumed any interocular difference in forces was likely to be both trivial and within the margin of error of the test procedure. One operator (P.N.) observed the right eye through the slit-lamp microscope and the second operator (C.K.) inserted the scleral contact lens with premounted magnet onto the patient's eye. The sliding arm, bearing an orbital part prototype at its tip, then was advanced until the 2 magnets were just in contact. At this point, suction was applied, transiently fixing the scleral contact lens to the eye for approximately 30 seconds, while the degree of ocular stabilization was observed through the slit-lamp microscope; during this period, the patient was asked to make a few saccades to check that he was able to overcome the magnetic force. The stabilization was repeated 3 times. A cylindrical N40 neodymium–iron–boron magnet of 4 mm in diameter and 2 mm in length was found both to stabilize the nystagmus and also to allow saccadic refixations. The ocular surface was inspected after the procedure to ensure that no abrasion had occurred.

### Implantation Surgical Procedures

**Figure 1:**
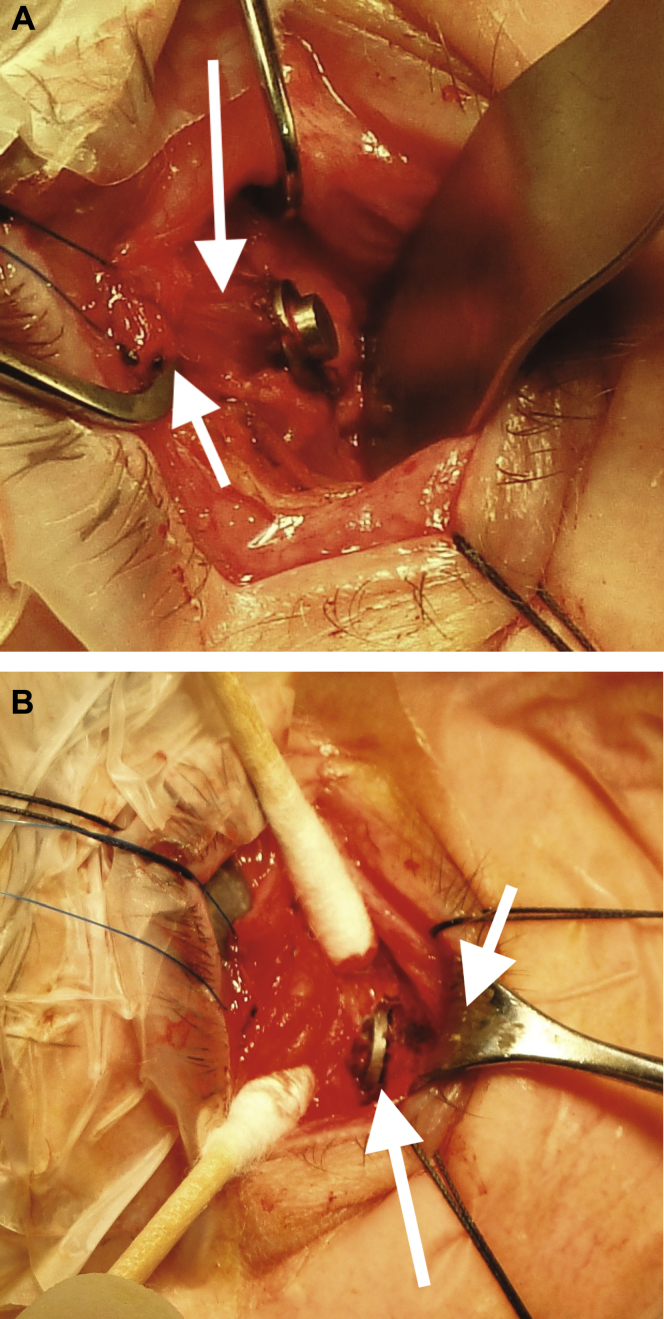
Photographs of right eye obtained during surgery showing: (**A**) the exposed inferior rectus muscle (*long arrow*) with a squint hook retracting the muscle at its insertion to the globe (*short arrow*) and (**B**) the positioned orbital magnet fixed to the orbital floor (*long arrow*) and the lower lid being retracted with a Desmarres retractor (*short arrow*).

Magnet implantation was performed under general anesthesia, with an interval of 4 months between the first (right) and second (left) orbits. The lower eyelid was retracted with 2 sutures of 2-0 silk, and the conjunctiva opened along its length in the depth of the fornix. With the conjunctiva placed on traction with 2 sutures of 6-0 silk, the anterior portion of the inferior rectus muscle was exposed by blunt dissection, and the Tenon's sheath was opened anteriorly and brushed backward to expose the anterior 15 mm of the inferior rectus. The smaller magnet (the ocular component) was sutured to the undersurface of the inferior rectus, centered approximately 10 mm behind the muscle insertion with 3 sutures of 6-0 polyglactin; the Tenon's sheath then was drawn forward over the magnet and sutured anteriorly with the same suture (Fig 1A). The orbital floor was exposed through the same incision, the larger magnet was placed alongside the ocular magnet, and the eye was aligned to primary position, allowing an estimate for the optimal position of the orbital magnet; this position was maintained while medical-grade cyanoacrylate glue was run into the gap between the orbital floor and magnet base (Fig 1B). The conjunctiva was closed with 1 rapidly absorbing 8-0 polyglactin suture, chloramphenicol ointment was instilled into the conjunctival sac, and the eye was padded for 48 hours. The patient was discharged home with a rapidly reducing course of oral corticosteroids, oral antibiotics, and topical chloramphenicol.

### Postimplantation Surgical Procedures

The patient continued to experience problematic diplopia, as not uncommonly observed in paraneoplastic cerebellar disorders,^11^, ^12^ with a moderate A-pattern esotropia. Seven months after implant surgery, the right esotropia reduced to 18 PD base out with 5 D hypertropia. An A pattern was still apparent, with mild bilateral lateral gaze defects (−0.5) and bilateral upgaze defect (−1). He underwent 3 strabismus procedures at 7, 20, and 25 months after orbital implantation: right eye adjustable medial rectus recess (inferior transposition) and lateral rectus resect (inferior transposition) for vertical element; bilateral superior oblique tenotomies; and left eye adjustable medial rectus recess and lateral rectus resect (no transposition), respectively. In the interval between the first 2 strabismus procedures, his symptoms were managed with Fresnel prisms, Bangerter occlusion foil, and botulinum toxin injections to both medial and inferior rectus muscles. After the third strabismus operation, the patient has maintained a useful central area of binocular single vision with regular botulinum toxin injections to the medial rectus muscle to manage his residual 16-PD base-out esotropia, because he remains intolerant of a prism of more than 12 D base out.

### Imaging

**Figure 2:**
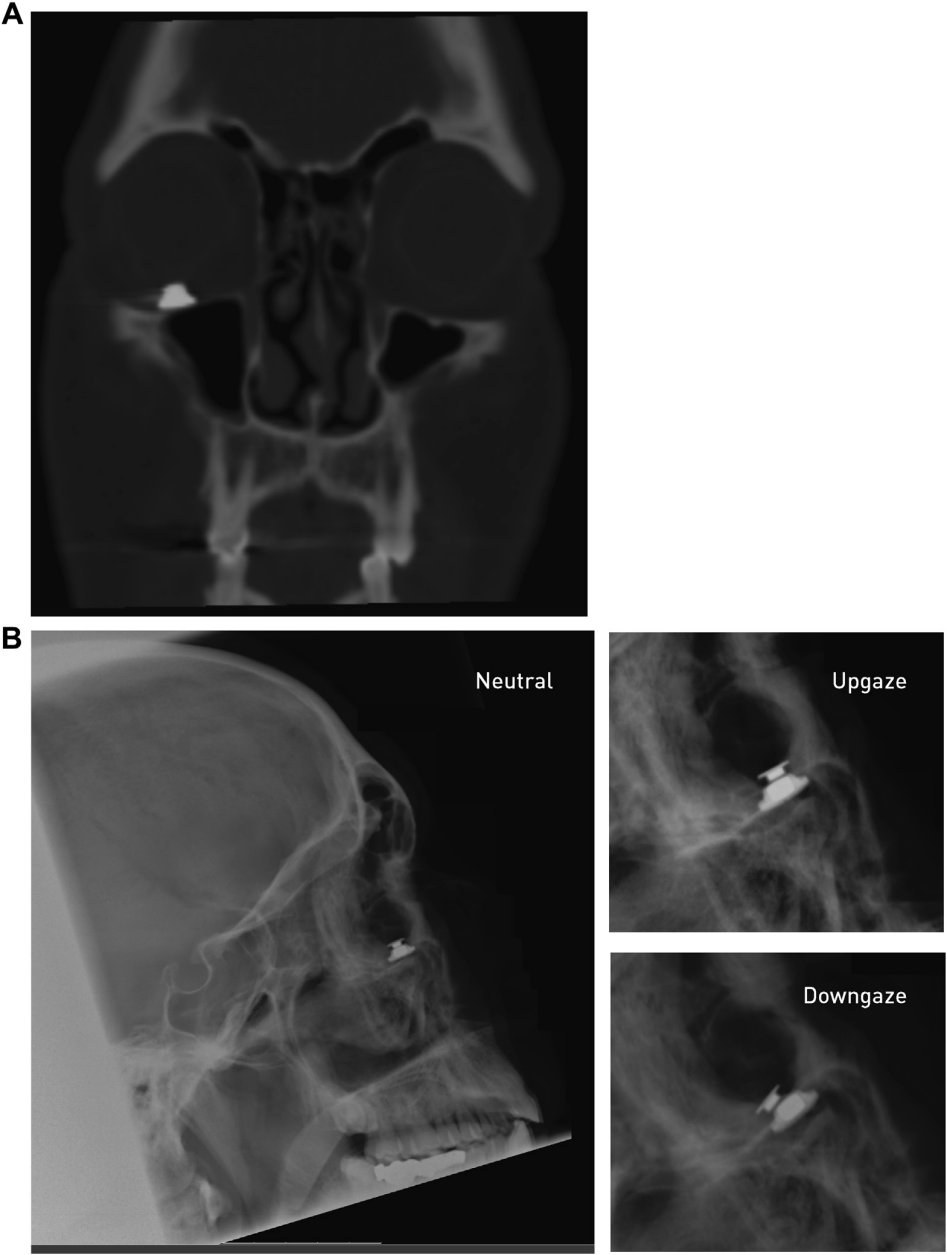
**A**, Orbital computer tomographic image showing the position of the right implant in the coronal plane. Note that the space between the 2 components is not visible because of artefact. **B**, Lateral plain radiograph showing the position of the right implant in the sagittal plane in 3 positions of gaze: neutral, upgaze, and downgaze. Note the movement of the eye component relative to the orbital component.

The orbit was imaged after the first implantation (Fig 2A); lateral plain radiographs in upgaze and downgaze also were obtained to show the relative excursion of the implant parts (Fig 2B).

### Role of the Funding Source

Apart from funding the project and monitoring its progress, the funder's role was limited to reviewing a draft of the article before submission.

## Results

After recovery from surgery, the patient reported that the oscillopsia had improved, especially on downgaze, although this was accompanied by a greater perception of diplopia, which had been a prominent symptom before oscillopsia developed. His visual acuity improved from 6/9 bilaterally to 6/6 on the left and 6/5–2 on the right, in the direction of optimally stabilized gaze; these parameters have remained stable over 4 years of follow-up.

The relative position of the 2 parts varied with the gaze, revealing the mechanism producing a gaze-dependent damping effect. Lateral radiographs show magnetic separation to be greatest on downgaze and accompanied by a tilt of the ocular part, causing the axis of maximum magnetic force and the axis of eye oscillation to coincide more closely. Therefore, the vector component contributing to the damping is increased compared with other positions of gaze (Fig 2B).

**Figure 3:**
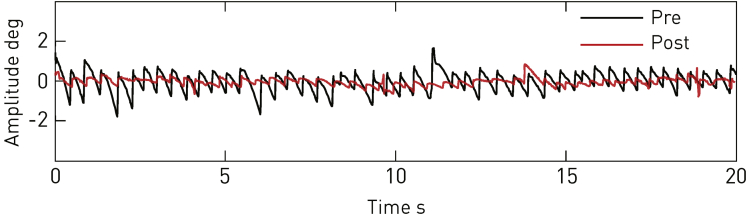
Example oculomotor trace before (Pre) and after (Post) surgery. A sample of eye movement amplitude during attempted fixation, indexed along the vertical displacement and plotted before (*black*) and after (*red*) bilateral implantation. The trace is derived from the left eye in a position of moderate damping: 15° downgaze in the vertical midline. Note the obvious fall in amplitude.

**Figure 4:**
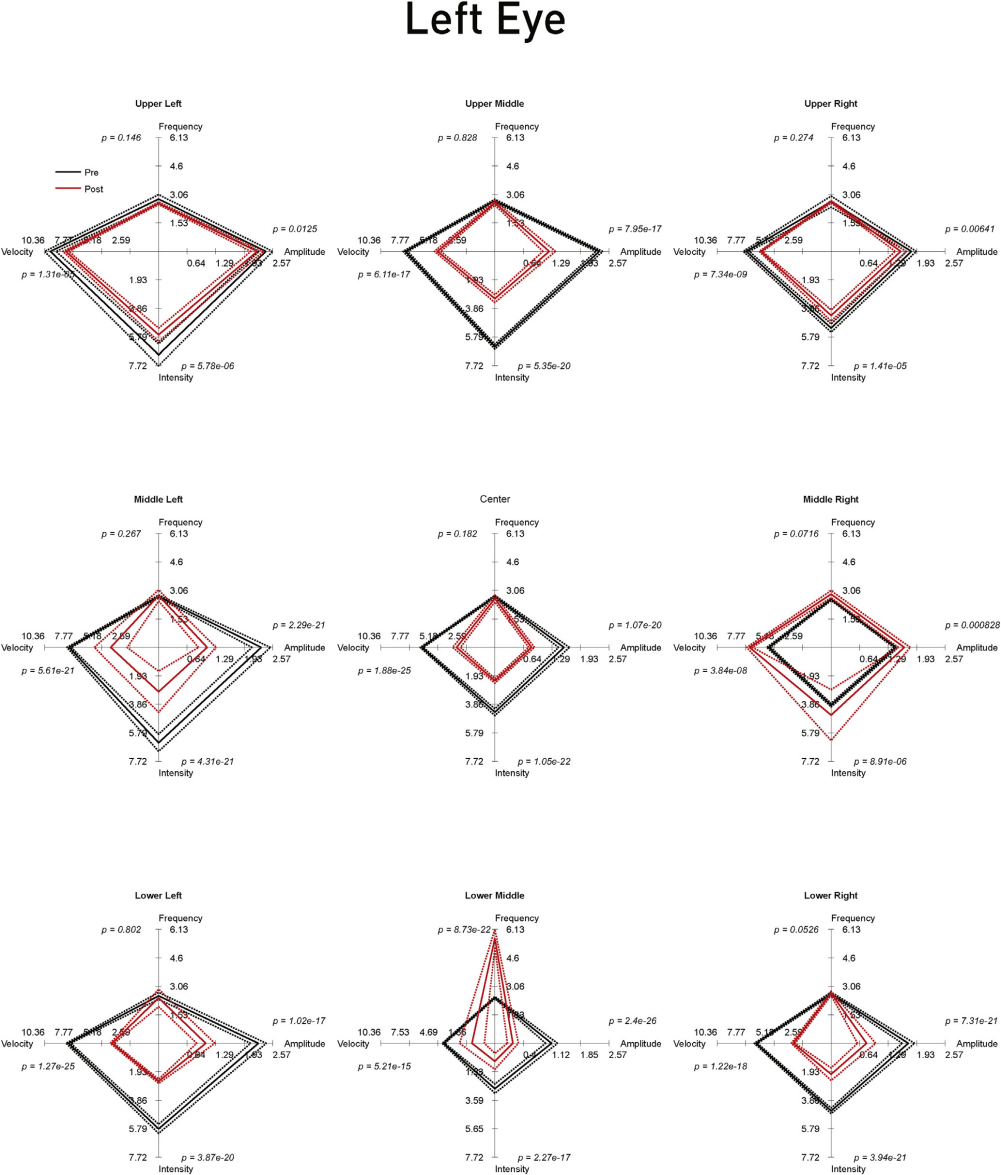
Spider plots of the nystagmus parameters in the left eye before and after bilateral surgery. Each of the panels corresponding to the 9 positions of evaluated gaze shows the median amplitude, frequency, presaccadic velocity, and intensity of the nystagmus in the left eye at that position before (Pre; *solid black line*) and after (Post; *solid red line*) bilateral surgery. The *dotted lines* show ±1 standard error of the median. The significance (*P*) values at each corner are derived asymptotically from a 2-sample Kolmogorov-Smirnov test for a difference in the paired distributions. The axes ranges are set to the maximum across eyes and conditions. On each of the 4 axes, a smaller value corresponds to a decrease in the corresponding nystagmus parameter: where the resultant diamond shape is smaller after implantation, the nystagmus is improved objectively. Note that there is generally a marked improvement in the parameters, especially in the downgaze positions as observed clinically.

**Figure 5:**
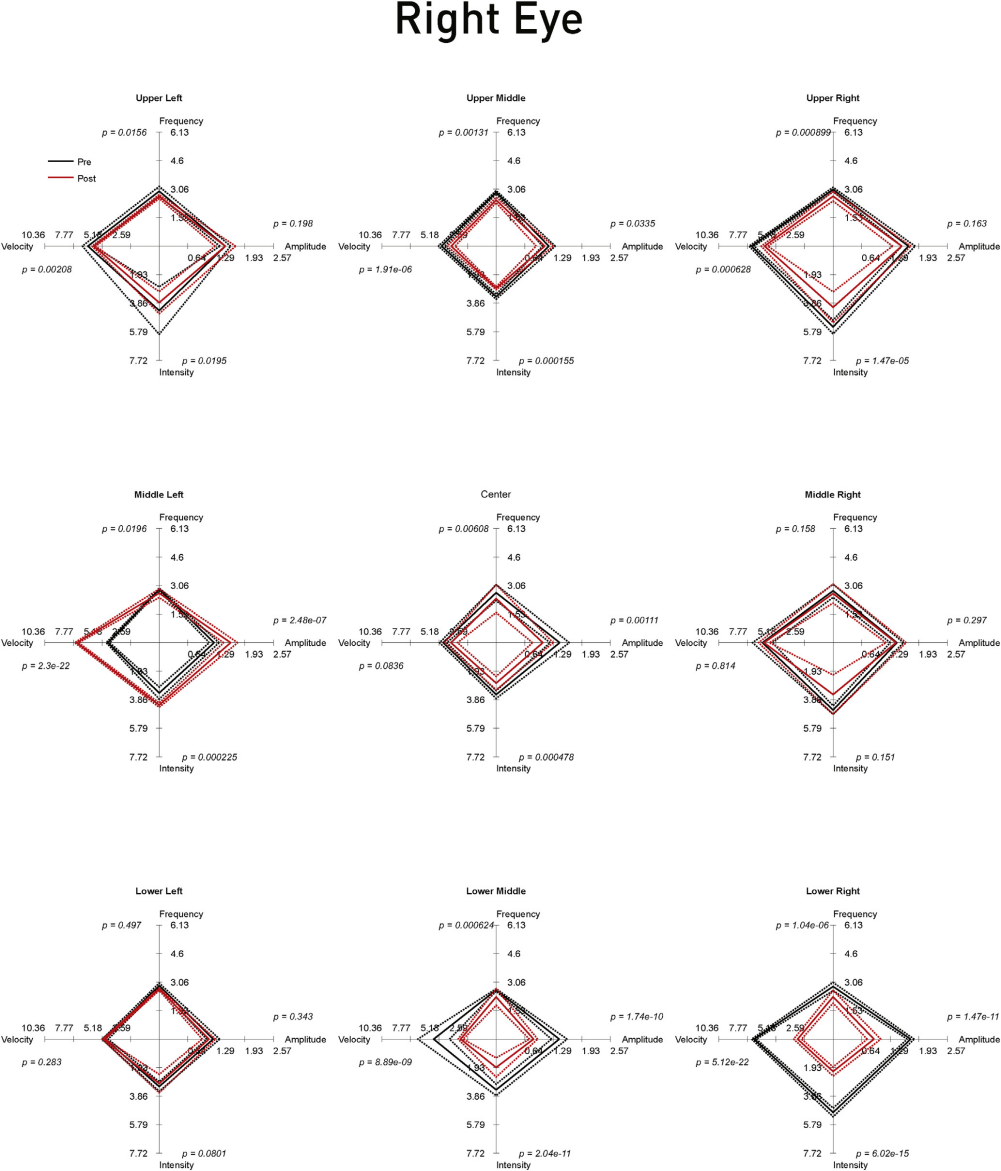
Spider plots of the nystagmus parameters in the right eye before and after bilateral surgery. Each of the panels corresponding to the 9 positions of evaluated gaze shows the median amplitude, frequency, presaccadic velocity, and intensity of the nystagmus in the right eye at that position before (*solid black line*) and after (*solid red line*) bilateral surgery. The *dotted lines* show ±1 standard error of the median. The significance (*P*) values at each corner are derived asymptotically from a 2-sample Kolmogorov-Smirnov test for a difference in the paired distributions. The axes ranges are set to the maximum across eyes and conditions. On each of the 4 axes, a smaller value corresponds to a decrease in the corresponding nystagmus parameter: where the resultant diamond shape is smaller after implantation, the nystagmus is improved objectively. Note that there is generally a marked improvement in the parameters, especially in the downgaze positions as observed clinically.

A quantitative assessment was carried out 8 days after the second implantation, and inspection of the raw traces showed an evident change from the preoperative state (Fig 3), this being reflected in the objective metrics of oculomotor behavior (Figs 4 and 5). The metrics considered most closely related to symptoms—amplitude and intensity—improved significantly in both eyes for most positions of the gaze, but especially on downgaze. The overall picture remained substantially unchanged with off-line recalibrated data (Table S1, available at www.aaojournal.org).

**Figure 6:**
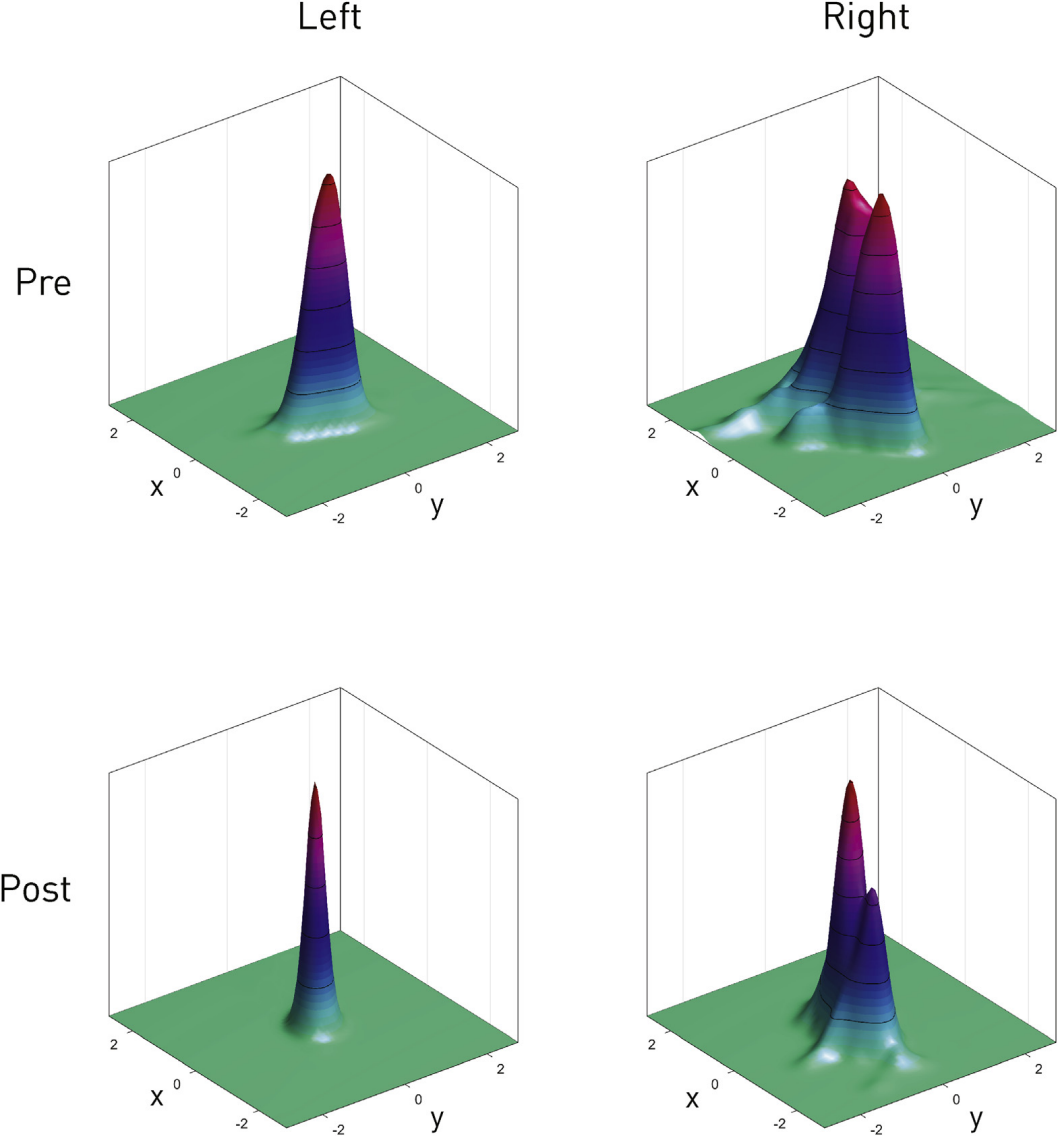
Visualization of the impact on fixation of the implantation. Plotted for each eye before and after surgery are fits of the spatial probability density functions of the point of gaze during fixation at the maximally damped position for each eye. The spatial scale is in degrees, identical in the *x*- and *y*-planes. The narrower the distribution is, the more stable the fixation is. Note the apparent improvement in the stability of fixation reflecting the change in nystagmus parameters shown in Figures 3 and 4.

To illustrate improved fixation, we further estimated the probability–density functions describing the variability in fixation position for the gaze locations with greatest damping. The resultant 2-dimensional surface plots (Fig 6) define the degree of fixation stability by the width of the visualized peak: the narrower the peak, the more stable the fixation. These subjective and objective improvements in vision were paralleled by real-life impact: the patient was able to return to part-time work, although not as a driver, and daily functions such as reading and watching television improved substantially. Nonetheless, he remains symptomatic from a relatively small degree of diplopia, correction of which is complicated by the fusional deficit (Fig S7, available at www.aaojournal.org). Importantly, there has been no discernible impact on shifts of gaze or on functional range of eye movement over 4 years.

## Discussion

This is the first description of the successful treatment of an oculomotor disorder with an implanted prosthesis, addressing a functional abnormality of neural origin by remote physical means. It opens the way to other applications where oculomotor dysfunction cannot be corrected by altering neural function and where the desired effect is beyond the reach of conventional surgical methods. Although a passive device was used, even more precise modification may be achievable with the use of active devices that are able to adapt dynamically and responsively to the host. Here we discuss the possibilities and limitations of the approach in relation to the treatment of nystagmus.

Although we remain far from a mechanistic explanation of nystagmus, the diversity of causative pathologic features suggests a mechanistic heterogeneity that is unlikely to be addressable by any simple manoeuvre at the neural level. Indeed, this is reflected in the clinical experience of the agents currently in use, where side effects may be as prominent as any—generally limited—therapeutic benefits. By focusing on the effector—the eye muscles themselves—the therapeutic approach can be insulated from its neural causation: What matters is the movement of the eye, not how it is generated.

Because the pathologic component of nystagmus is the slow drift that the fast phase corrects subsequently, adequate damping can be produced by a force substantially smaller than that involved in the generation even of relatively small saccades. This minimizes the risk of immobilizing the eye completely and allows the implant to be small and straightforward to insert. By inserting the ocular part inside Tenon's sheath, thereby maintaining a lubricated sliding surface between the parts of the implant, fibrosis that otherwise could restrict eye movement probably can be avoided.

The procedure is relatively straightforward, does not require intraoperative imaging, and relies on no specialist equipment other than nonmagnetic surgical instruments. The implant itself is relatively inexpensive to manufacture, and the use of titanium—the only structural material in contact with the body—is well established in the orbit. The patient made a rapid recovery from both implantations, with slightly more inflammation after the first surgery when postoperative steroids were not administered. Both subjectively and objectively, the patient's visual function improved, and this was reflected in real-life changes in his capacity for employment. However, the ocular stabilization did reveal problems with binocular fusion that had been present before surgery.

There are, however, some limitations that need careful consideration. Whether the benefits can be extended to other types of nystagmus remains to be seen. Because the presence of ferromagnetic materials is an absolute contraindication to magnetic resonance imaging, patients with progressive or recurrent neurologic illness requiring magnetic resonance surveillance are not candidates for such surgery. It is also worth noting that the strongest effect observed in our patient was the creation of a downgaze null point, and it may be the case that only patients without a natural null point are likely to experience a similar functional benefit. Finally, any changes of extraocular muscle function risk creating or exacerbating binocular diplopia. Owing to cerebellar syndrome, there was pre-existing diplopia resulting from a fusional defect, and this became more evident and symptomatic after surgery. Where more than 1 set of implants are used or where the implant is in the lateral plane, such effects may be less pronounced.
