# Supplementary material for: Magnetic Oculomotor Prosthetics for Acquired Nystagmus
Source: Ophthalmology. 2017 Oct;124(10):1556–64. doi: 10.1016/j.ophtha.2017.05.028 (PMC5609850; doi:10.1016/j.ophtha.2017.05.028)
Supplement: Fig S7 [file mmc1.pdf]

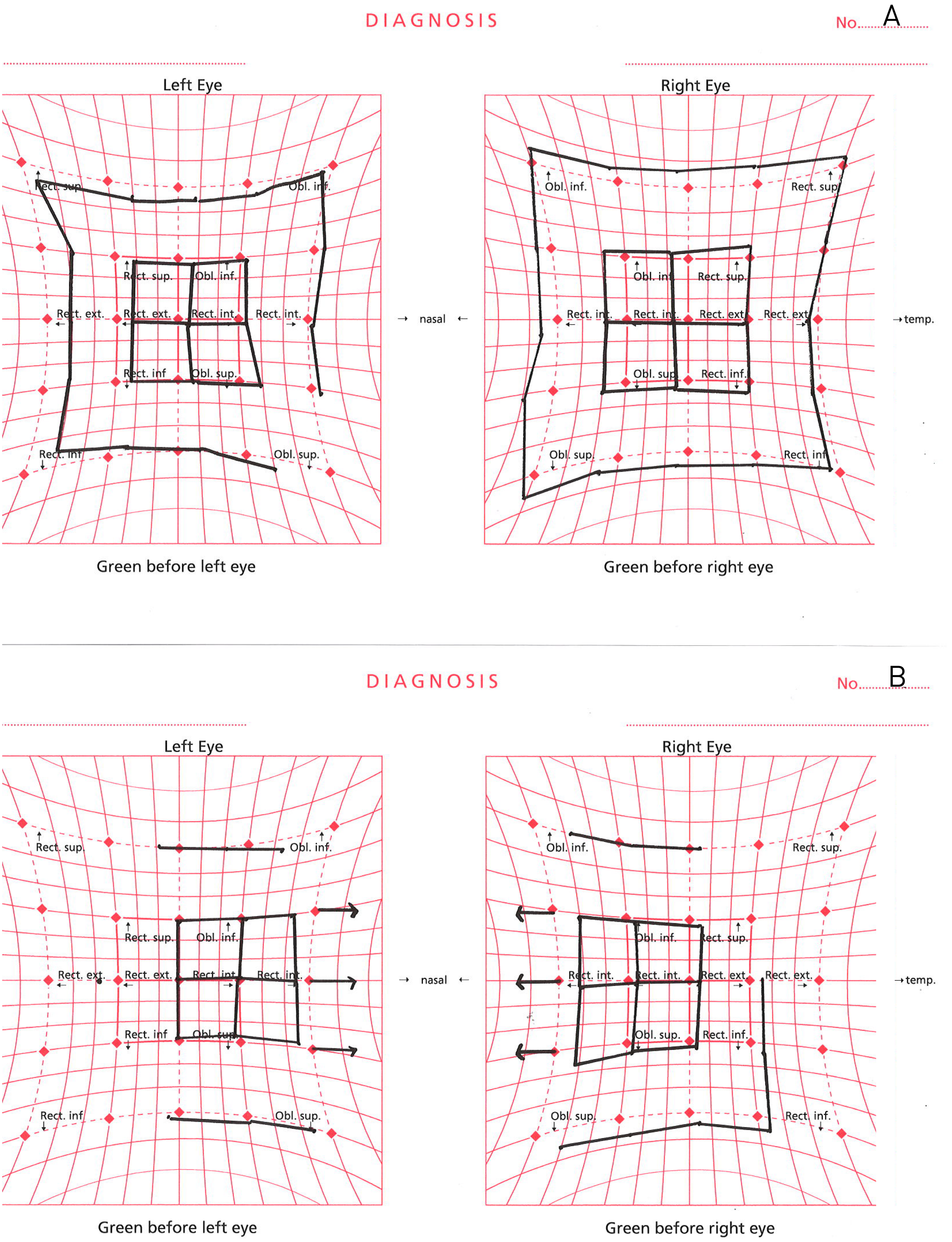

Figure 7. Hess charts at presentation (A) and 12 months after symptom onset (B), both prior to implantation.
