# Supplementary material for: Magnetic Oculomotor Prosthetics for Acquired Nystagmus
Source: Ophthalmology. 2017 Oct;124(10):1556–64. doi: 10.1016/j.ophtha.2017.05.028 (PMC5609850; doi:10.1016/j.ophtha.2017.05.028)
Supplement: Table S1 [file mmc2.pdf]

Left eye

UL amplitude	UL velocity	UM amplitude	UM velocity	UR amplitude	UR velocity
**p=0.0822**	p<0.0001	p<0.0001	p<0.0001	p=0.0185	p<0.0001
ML amplitude	ML velocity	C amplitude	C velocity	MR amplitude	MR velocity
p<0.0001	p<0.0001	p<0.0001	p<0.0001	p<0.0001	p<0.0001
LL amplitude	LL velocity	LM amplitude	LM velocity	LR amplitude	LR velocity
p<0.0001	p<0.0001	p<0.0001	p<0.0001	p<0.0001	p<0.0001

Right eye

UL amplitude	UL velocity	UM amplitude	UM velocity	UR amplitude	UR velocity
p=0.2955	p=0.0114	**p=0.1673**	p<0.0001	p=0.3579	p=0.0012
ML amplitude	ML velocity	C amplitude	C velocity	MR amplitude	MR velocity
p<0.0001	p<0.0001	**p=0.0557**	p=0.2284	p=0.5371	p=0.9662
LL amplitude	LL velocity	LM amplitude	LM velocity	LR amplitude	LR velocity
p=0.1242	**p<0.0001**	p<0.0001	p<0.0001	p<0.0001	p<0.0001

Table 1. Statistical re-analysis of amplitude and drift velocity following off-line calibration correction. On-line calibration achieved reasonable calibration accuracy as indexed by the distance between the mean fixation position estimated by the calibration routine and the actual target position, averaged over the 9 locations. These values were as follows: Left pre-op: 0.6002 degrees, Right pre-op: 2.3485 degrees, Left post-op: 0.8427 degrees, Right post-op: 0.6514 degrees. Since our calibration targets were far apart, forming a 31 degree square, a 2.4 degree error corresponds to a maximum possible scaling error of 7.7%: a very small change in comparison with the observed effects. Here we nonetheless give the results of a re-analysis, where the original eye position data has been subjected to an affine transform (6 parameters), derived by minimizing the squared error over possible transformation matrices, that takes into account translational, rotational, skew, and scaling differences between calibrated and actual positions. Since all such corrections inevitably assume a linear interpolation of error across locations, unjustifiably propagating errors across the field of view, we present these results in addition to, rather than instead of, the original data. Note that from 36 comparisons only 4 materially change (in bold), three becoming no longer statistically significant and the fourth becoming newly significant, leaving the overall picture essentially identical. Abbreviations: UL-upper left, UM-upper middle, UR-upper right, ML-middle left, C-centre, MR-middle right, LL-lower left, LM-lower middle, LR-lower right.
